# Variation of the Pore Morphology during the Early Age in Plain and Fiber-Reinforced High-Performance Concrete under Moisture-Saturated Curing

**DOI:** 10.3390/ma12060975

**Published:** 2019-03-24

**Authors:** Miguel A. Vicente, Jesús Mínguez, Dorys C. González

**Affiliations:** 1 Department of Civil Engineering, University of Burgos, c/Villadiego, s/n, 09001 Burgos, Spain; jminguez@ubu.es (J.M.); dgonzalez@ubu.es (D.C.G.); 2 Parks College of Engineering, Aviation & Technology, Saint Louis University, 3450 Lindell Blvd, Saint Louis, MO 63103, USA

**Keywords:** pore morphology, voids, fiber-reinforced concrete, CT scan technology, DIP software

## Abstract

In this paper, two concrete mixtures of plain concrete (PC) and steel fiber-reinforced high-performance concrete (SFRC) have been scanned in order to analyze the variation of the pore morphology during the first curing week. Six cylinders of 45.2-mm diameter 50-mm height were performed. All of the specimens were kept in a curing room at 20 °C and 100% humidity. A computed tomography (CT) scan was used to observe the internal voids of the mixtures, and the data were analyzed using digital image processing (DIP) software, which identified and isolated each individual void in addition to extracting all of their geometrical parameters. The results revealed that the SFRC specimens showed a greater porosity than the PC ones. Moreover, the porosity increased over time in the case of SFRC, while it remained almost constant in the case of PC. The porosity increased with the depth in all cases, and the lowest porosity was observed in the upper layer of the specimens, which is the one that was in contact with the air. The analysis of the results showed that the fibers provided additional stiffness to the cement paste, which was especially noticeable during this first curing week, resulting in an increasing of the volume of the voids and the pore size, as well as a reduction in the shape factor of the voids, among other effects.

## 1. Introduction

Concrete is a porous material in nature. The content of pores is very wide between different concrete mixtures. In most cases, the concrete elements show an inevitable porosity, which is what remains inside concrete mixtures after being vibrated to eliminate all the entrained air (using, for example, surface or needle vibrators, etc.). In other cases, the concrete mixture is specially designed to include a certain porosity level, depending on particular needs.

Porosity has a strong influence on the behavior of fresh concrete, because it modifies the rheology of the mixture [1,2,3]. Moreover, porosity modifies the mechanical properties and the durability of hardened concrete. Among other effects, porosity has a relevant impact on permeability [4,5,6,7], the behavior under freeze-thaw cycles [8,9], the behavior under fire [10], and the behavior under fatigue loading [11,12,13].

The porosity of concrete must not necessarily be a problem. In fact, in some cases, porosity is a sought property. For example, an increase in porosity leads to an increase in the flowability of concrete, which is a needed requisite for pumpable concretes [14]. Pervious concrete is a good example of how porosity is a sought-after property. In this case, concrete pavement is designed with an extremely high volume of pores, in order to assure that roads remain dry when it is raining, even during extreme downpour, increasing the safety of the road [4,6]. This same idea can be used for ultralight concretes [15].

Another situation where air voids are useful is in the case of concrete elements subjected to freeze–thaw cycles [8,9]. Under these cycles, porosity improves the resistance of the concrete. In fact, concrete elements placed in regions with extreme freeze–thaw cycles events must be designed with a minimum threshold of porosity.

On the other hand, there has been an increasing use of fiber-reinforced concrete. It is a very suggestive solution because of the reduction of the labor cost, especially if it is combined with self-compacting concrete. In most cases, fibers are used to improve the mechanical behavior of concrete: they reduce cracking [16,17,18], improve the fatigue life [19,20,21,22], increase the tension strength capacity of concrete [23,24], improve the behavior under freeze–thaw cycles [25,26], and extend the fatigue life [27,28].

A different case involved the use of plastic fibers to improve the behavior of concrete under fire [29,30]. In this case, the strategy is that plastic fibers melt under a relatively low temperature, which is significantly below the temperature that fire typically starts to result in spalling in conventional concrete, and thus the internal overpressure caused by water vapor is dissipated.

However, in all the research works mentioned above, it has been implicitly assumed that fibers do not modify the concrete matrix, i.e., the microstructure of concrete matrix and, in particular, the pore morphology (voids content, pore size distribution, shape of the voids, etc.) is not affected by the presence of fibers.

The voids inside concrete can be classified into micropores (size less than 1 μm), mesopores (size between 1–10 mm), and macropores (size greater than 10 mm) [31]. Several methods can be found in the literature to analyze the pore structure. The traditional ones are nitrogen absorption and mercury intrusion porosimetry (MIP) [32,33]. These methods show two main limitations. First, they can only provide the pore size distribution, but not the pore distribution, shape, etc. Second, these techniques can only provide information about open porosity, and not about closed porosity.

Currently, the use of computed tomography (CT) scan technology is being used to analyze, in general, the microstructure of concrete and, in particular, the pore structure. Most of the research conducted has focused on fiber-reinforced concrete, and hence fiber orientation [12,34,35]. However, in the last years, there has been a growing interest in the internal pore structure, and several works have been published in this area [4,36,37,38,39,40,41,42,43].

Using CT scan technology, it is possible to visualize all the pores of the concrete samples, and not only the open porosity, but also the closed porosity. CT scan technology provides a lot of useful information of each individual void, such as the position, volume, length, etc. With this information, it is possible to determine several geometrical parameters, such as the shape factor, among others. Moreover, it is possible to obtain several correlations, such as the spatial distribution, among others.

In addition, when the scanning process is carried out daily during the first curing week of the specimens, it is possible to analyze the evolution of all the geometrical parameters of the voids over time.

This information can be used as a basis to establish the correlation between the porosity of the concrete and its macroscopic response.

The CT scan technology is a powerful tool; to date, no other technology can provide this information about the internal microstructure of concrete.

In this paper, the CT scan is used in order to detect the voids inside two different concrete mixtures: plain and fiber-reinforced concrete, and also to study the evolution of the voids over time, during the first curing week. In both cases, the concrete paste is the same, and the only difference is the presence of steel fibers. Using post-processing routines especially developed by the authors, it is possible to analyze the pore morphology in both cases: porosity, pore size distribution, pore shape, etc., and its variations with time during the early ages of concrete. The results show that plain and fiber-reinforced concrete mixtures have initial differences in pore morphology, and they also exhibit a different variation with time. The final result is that both concretes have very different pore morphology at the end of the studied time. The results also reveal the two mechanisms behind the differences between the final pore morphology of plain and steel fiber-reinforced concrete.

This paper is structured as follows: the experimental procedure is presented in Section 2, the results of the tests are described and discussed in Section 3, and finally the conclusions are found in Section 4.

## 2. Experimental Program

In this section, the materials, the manufacturing procedure, and the scanning procedure are described.

### 2.1. Materials

A total of six cylinders that were 45.2 mm in diameter 50 mm in height were manufactured. Three of them were of plain high-performance concrete (PC), and the rest were steel fiber-reinforced high-performance concrete (SFRC). The mixing proportions by weight, in both cases, were: 1:2.00:0.015:0.31:0.035 (cement:fine aggregate:nanosilica:water:superplasticizer). The fiber-reinforced concrete was reinforced with 7.8 kg/m^3^ of steel fibers Dramix OL 8/.16 (BEKAERT, Kortrijk, Belgium), which means that concrete had a fiber volume fraction of 0.1%. Fibers were 8 mm in length and 0.16 mm in diameter (aspect ratio 50). According to the technical information provided by the supplier, their tensile strength was 3000 MPa, and their modulus of elasticity was 200 GPa. The nanosilica used was MasterRoc MS 685 (BASF, Ludwigshafen am Rhein, Germany). The superplasticizer used was Glenium 52 (BASF, Ludwigshafen am Rhein, Germany). Siliceous aggregate was used, with a nominal maximum aggregate size of 4 mm. Portland cement with a high initial strength of CEM I 52.5 R was used.

Additionally, two prisms that were 40 × 40 × 160 mm were manufactured; one per mixture. A total of three cubes with 40-mm edges were obtained from each prism, and they were tested under compression in order to characterize the compression strength (according to EN 196-1:2016). The concrete compressive strength f_c_’ was 68.9 MPa with a standard deviation of 1.9 MPa.

### 2.2. Specimen Manufacturing Process

**Figure 1:**
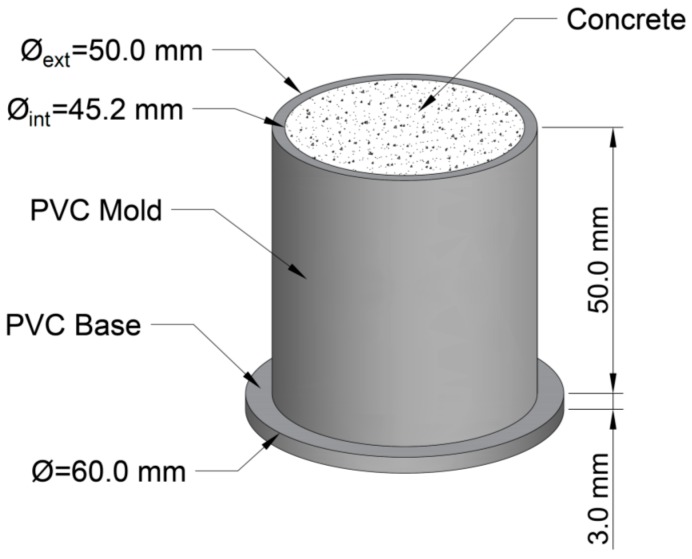
Mold and specimen.

The six cylinders above mentioned were cast in the same number of Polyvinyl chloride (PVC) molds with a 45.2-mm inner diameter, 50-mm outer diameter, and 50-mm height. At the bottom of each pipe, a PVC disc with a 60-mm diameter and 3-mm thickness was welded, in order to ensure a watertight joint (Figure 1). The concrete was built using a cement mortar mixer, and the manufacturing process followed the standard EN 196-1:2016 [44]. The molds were filled in two parts using a small aluminum scoop to form the specimens without applying vibration. However, some small punches were applied on the side of the molds, once it was filled with mortar, to help the mortar expel the entrapped air. The upper surface was smoothed with a trowel. Once all of the specimens were cast, they were kept in a moisture curing room, where they remained at 20 °C and 100% humidity.

### 2.3. Scanning Process

**Figure 2:**
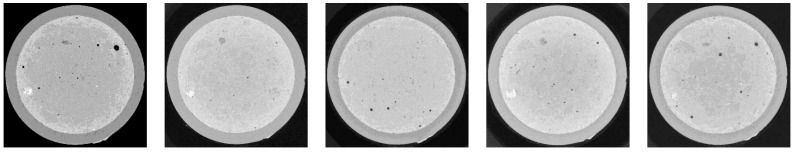
Slices belonging to plain concrete at different ages.

**Figure 3:**
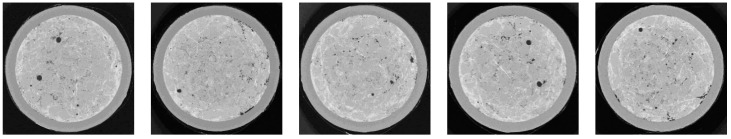
Slices belonging to steel fiber-reinforced concrete at different ages.

During the first week, a total of five scans were performed of each specimen, belonging to the following ages: one day, two days, three days, four days, and seven days. The CT scan used was a GE Phoenix v|tome|x device (General Electric, Boston, MA, USA), belonging to the ‘Centro Nacional de Investigación sobre la Evolución Humana (CENIEH)’, in Burgos, Spain. It was equipped with a tube of 300 kV/500 W. This facility emits a cone ray, which is received by an array of detectors. Thereby, the scanning process is fast, and highly accurate scans are produced of equal resolution in the X, Y, and Z axes. The rotation step around the Z-axis was 0.5°, so 720 shots were carried out on each specimen. The CT scan has a post-processing software that provides flat pictures of 2048 × 2048 pixels. Thus, for a section of 45.2-mm diameter, the equipment provides a horizontal resolution of 25 × 25 μm^2^. The vertical distance between the cutting planes was fixed at 25 μm, so the CT scan produced 2000 pictures per specimen, such as the ones shown in Figure 2 and Figure 3. The software assigns a grey level to each voxel (volumetric pixel), varying from zero to 255, where zero means black, and 255 means white, depending on the current density of the matter. Light grey voxels correspond to more dense points and dark grey voxels correspond to less dense points, i.e., pores. The final result of the CT scan is a matrix including X, Y, and Z coordinates of the voxel center of gravity and a number, from zero to 255, regarding the density. The total number of voxels in a specimen is approximately 4.3 × 10^9^. The average time of scanning is around 1 h for each specimen, including the post-processing process. A more detailed explanation of the CT scan technology and the scanning process can be found in [40,41,42,43,44,45].

**Figure 4:**
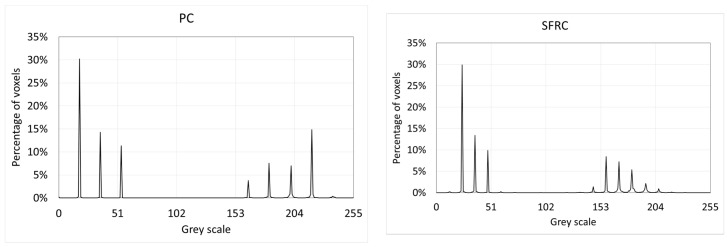
Histograms of grey scale.

Next, the digital image processing (DIP) software AVIZO (FEI Visualization Sciences Group, Hillsboro, OR, USA) was used to identify and isolate each individual void inside the specimen. First, the software identified the voxels belonging to voids, which are the ones showing a grey color below a threshold. In this case, once the histogram of grey distribution was studied, a threshold of 65 was considered (Figure 4).

Histograms revealed that the images have a great amount of dark-grey voxels. Most of them belonged to the empty space placed outside the mold. So, the next step was to delete all the voxels placed outside the inner face of the mold, because they did not belong to the concrete specimen. Moreover, the histograms of Figure 4 are not valid to obtain the percentage of voids in the specimen.

**Figure 5:**
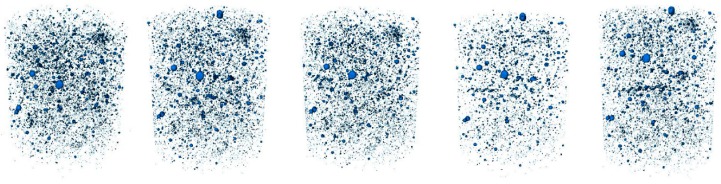
Three-dimensional (3D) views of the voids belonging to plain concrete at different ages. Age increases from **left** to **right**.

**Figure 6:**
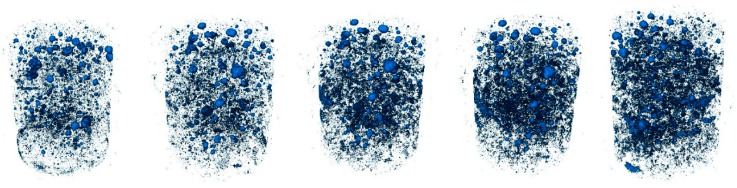
3D views of the voids belonging to steel fiber-reinforced concrete at different ages. Age increases from **left** to **right**.

Then, all the voxels in contact were merged, since they belong to the same void. The software identified and isolated the different voids. The final result of the scanning was a dot matrix containing the Cartesian coordinates X, Y, and Z of the center of gravity of each individual void. Figure 5 and Figure 6 shows the view of one specimen of each mixture along the timeline.

**Figure 7:**
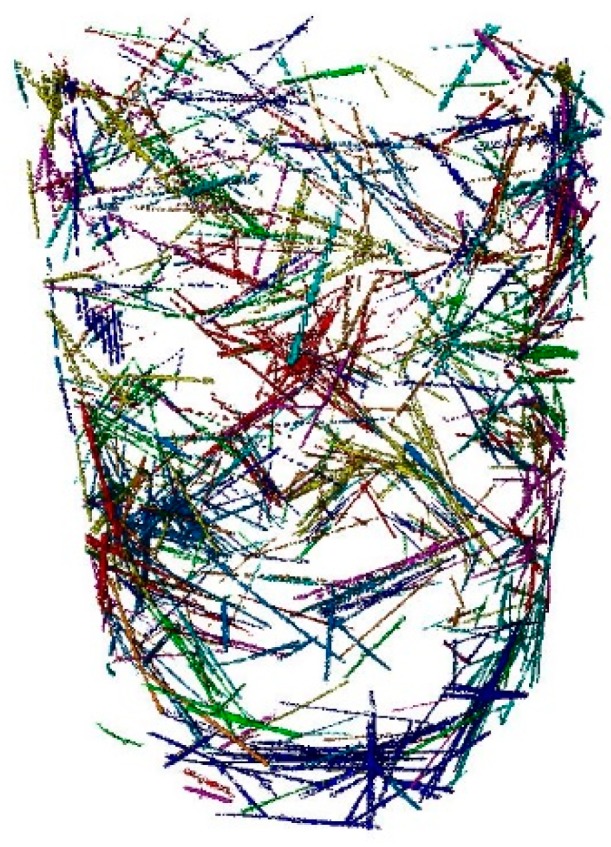
3D view of the fibers belonging to steel fiber-reinforced concrete.

Moreover, in the case of SFRC, the fiber distribution and orientation were obtained (Figure 7). In this case, the fibers did not move over time.

It can be noticed that a relevant part of the big pores was placed in the region that came in contact with the mold. This is because of the following: first, the concrete showed a wall effect, which led to a greater percentage of the smaller components of the cement paste (filler, cement, and water) in the lateral border. Second, the shrinkage of concrete resulted in a gap between the concrete specimen and molds. This space is initially filled with water and later with air, which can initially be understood as voids, although they cannot be considered ‘conventional’ voids.

**Figure 8:**
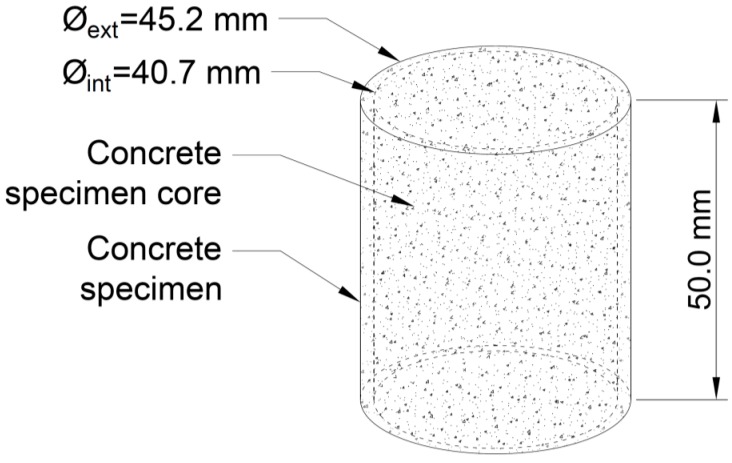
Portion of the concrete to be studied.

In order to prevent the distortion on the results that can be caused by the voids located in the lateral border of the cylinders, because of the reasons mentioned above, this lateral area was discarded. In particular, the whole cylinder was not studied; instead, only the inner portion of the cylinder was studied, i.e., a cylinder with a diameter that was 90% of the real diameter of the cylinder, i.e., 40.7 mm and 90% of the whole height, i.e., 50 mm (Figure 8).

In this work, voids with less than three voxels in the largest direction (i.e., 75-μm length, approximately) have been discarded because they are too small, and the CT scan does not provide enough sharpness to clearly identify them. Additionally, pores larger than 10 mm in the largest direction have been discarded, since they are non-representative pores. The results shown in this paper are the average values of the three specimens.

## 3. Results and Discussion

Using the naked eye, it is not possible to detect significant differences between the specimens and mixtures, nor the evolution of the specimens with time. Instead, digital image processing (DIP) software needs to be used in combination with homemade routines to deeply analyze the data and extract relevant information. Next, this in-depth analysis is shown, and some interesting conclusions are exposed. The values that are presented correspond to the average of the three specimens belonging to the same mixture.

### 3.1. Total Volume of Voids and Porosity

The first studied parameter is the total volume of the voids and the porosity; this last parameter is defined as the ratio between the volume of voids and the volume of the specimen.

**Figure 9:**
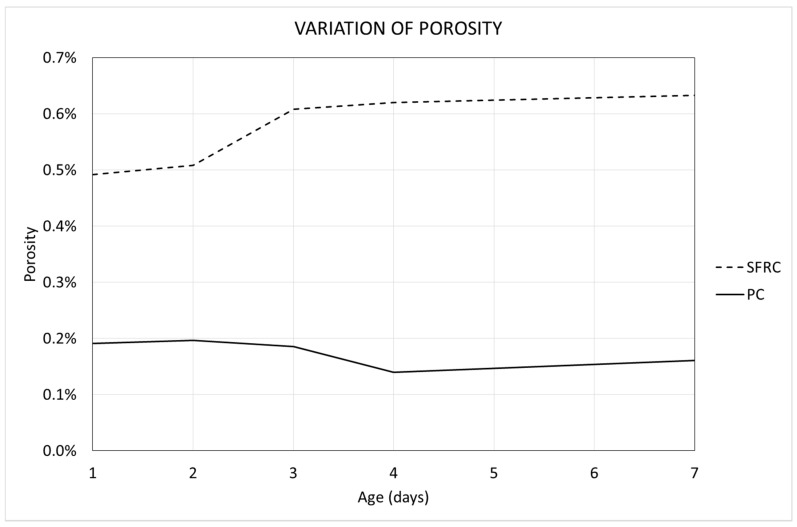
Variation of the porosity with the age of concrete.

Figure 9 shows the variation of the porosity with the age of concrete in the different mixtures.

Figure 9 shows some interesting conclusions. The first and most striking conclusion is that the porosity is greater in steel fiber-reinforced concrete (SFRC) than in plain concrete (PC). It is concluded that the fibers provoke an increase of the content of entrained air. It is known that the addition of steel fibers to the concrete mixture results in a modification of the properties of fresh concrete, which includes its viscosity, consistency, and porosity, among other properties. To avoid this, usually the concrete mix is corrected a bit in order to mitigate these effects. However, the aim of this work is to analyze how the addition of fibers modifies the evolution of porosity over time, during the first curing week, in the same concrete matrix.

The mixture used in this research is very fluid and, in both cases, a low porosity is observed. This result agrees with the ones obtained in other research, where it is demonstrated that fibers reduce the flowability of the mixture [46,47], hindering the removal of entrained air, and leading to a greater porosity.

Regarding the variation of porosity with time, it can be noticed that SFRC specimens show an increase of the porosity along a seven-day timeline. This is a damped process, since the variation is more intense during the first days, and it decreases with time. In the case of PC, the porosity shows a slight decrease over time.

The porosity of concrete is a dynamic phenomenon and two opposite forces are acting on it. First, the curing process is a water-consuming process. At the beginning of this process, most of the voids are full of water, and the CT scan does not recognize them as voids, since they are not air voids. Over time, because of the hydration process, water is reacting with cement grains to create hydration products. Progressively, water is being consumed and the space is occupied by the air, creating internal voids. The consequence is a progressive increase of the porosity in concrete.

Second, the air entrained inside the pores tends to move up and leave the concrete specimen. In addition, this space tends to be occupied by the cement paste when a collapse of the voids occurs. This second phenomenon implies a progressive decrease of the porosity in concrete. From the macroscopic point of view, this phenomenon is called plastic settlement or initial plastic shrinkage.

The SFRC showed a concrete matrix more consistent than that of the PC because of the presence of fiber. Consequently, in the case of SFRC, the first phenomenon prevails over the second one, so the final result is an overall progressive increase of the concrete’s porosity. On the contrary, in the case of PC, both phenomena are balanced, and the final result is that the porosity shows a very slight variation [48].

### 3.2. Variation of the Total Porosity along the Depth

**Figure 10:**
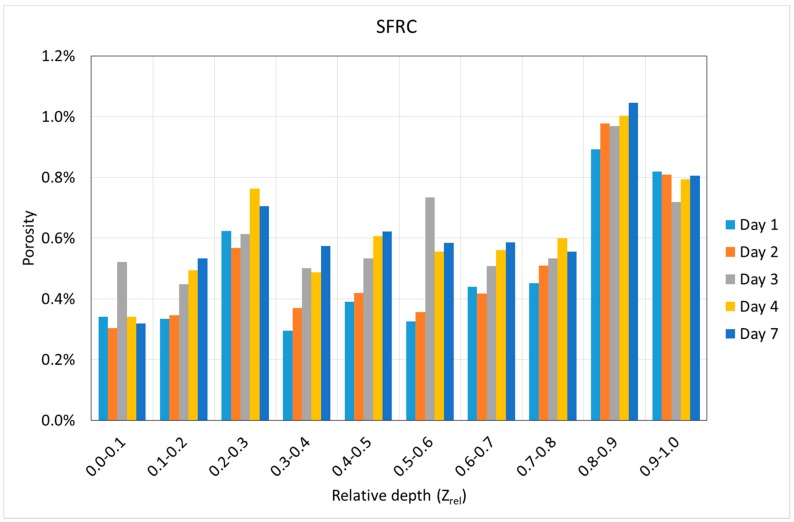
Variation of the porosity in steel fiber-reinforced high-performance concrete (SFRC) according to the depth and age.

**Figure 11:**
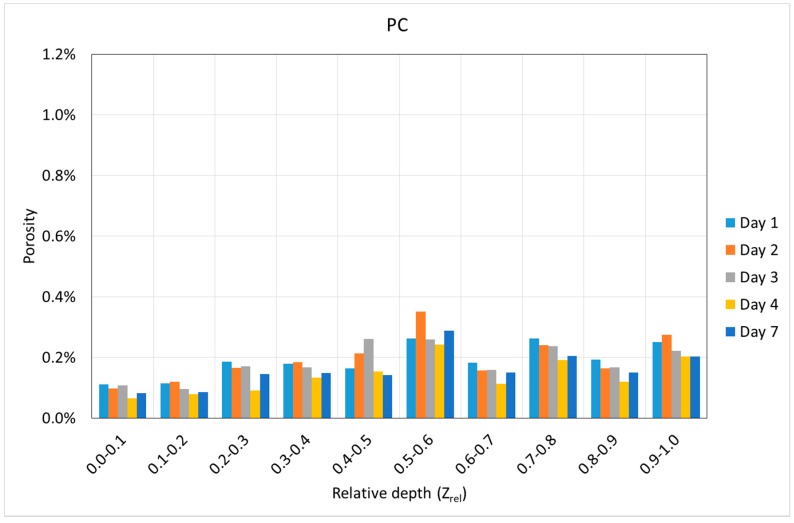
Variation of the porosity in plain concrete (PC) according to the depth and age.

Using the Z coordinate of the center of gravity of each void, it is possible to know the variation of the porosity along the depth. Figure 10 and Figure 11 show the variation of the porosity along the depth with the age of concrete in both mixtures. In all cases, the depth is shown in relative terms, i.e., varying from zero to one, where zero belongs to the exposed surface of the specimens, and one belongs to the other end of the specimens.

Figure 10 and Figure 11 show some interesting results. First, it can be noticed that a progressive increase of the porosity with the depth was observed in all cases. This is especially clear in the case of SFRC, but this tendency is not so clear in the case of PC. However, in both cases, the lowest porosity was observed in the first two-tenths of the specimens.

Second, SFRC showed a progressive increase of the porosity with time for all the depths, except for the 10% upper depth, where a slight decrease was observed. In the case of PC, a weak tendency of the porosity with time was observed; if anything, a slight decrease was also observed with time.

**Figure 12:**
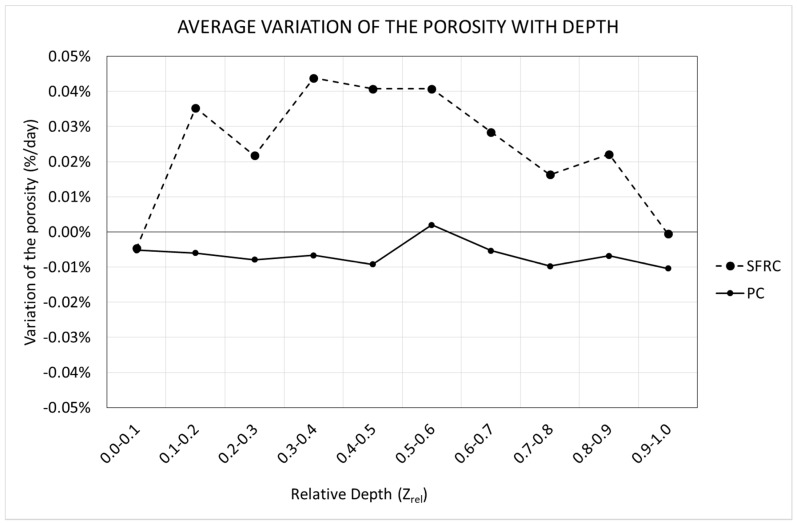
Variation of the porosity variation speed along the depth.

Using the data of the porosity with time, the best fitting line was obtained at each depth, and its slope was extracted. This represents the average value of the porosity variation speed during the first seven days. Figure 12 shows the variation of this parameter along the depth for both mixtures.

Figure 12 reveals that SFRC specimens showed a progressive increase of the porosity with time for almost all of the depths, except for the upper tenth, where a slight decrease was observed. In the case of PC, a slight decrease was observed for almost all the depths.

Again, the two different mechanisms regarding the void generation can be used to explain the behavior shown in Figure 12. Inside the specimen, when it is not close to the exposed surface, the loss of water is mainly due to hydration. In this case, the progressive reduction of free water, and in consequence, the progressive appearance of voids is strongly related to the creation of the cement matrix. In the case of SFRC, fibers provide extra stiffness to the cement matrix, which prevent the collapse of the voids. On the contrary, the cement matrix of PC specimens is less stiff, and the collapse of the voids happens more easily.

On the other side, close to the exposed surface, the loss of water is mainly due to evaporation, and the risk of a collapsing void increases. In this case, since the specimens are kept in a moisture curing room, the evaporation is almost null, and in consequence, this phenomenon can be observed only really close to the exposed surface.

In the case of SFRC, the average porosity variation speed is approximately 0.02%/day. On the contrary, in the case of PC, the average porosity variation speed is approximately −0.01%/day.

In all cases, the specimens show a slight different behavior around their middle height, i.e., at a relative depth of approximately 0.5. This is because the molds were filled in twice, and a horizontal joint appeared.

### 3.3. Porosity and Porosimetric Curves

Using the DIP software, it is possible to know the exact geometry of each void. At this point, it is interesting to obtain the volume of each void and its length, which is defined as the maximum distance between two voxels belonging to the same void.

Using this information, it is possible to obtain the pore volume curves and the porosimetric curves. A pore volume curve is defined as the graph correlating the length of the void and the total pore volume of the voids with a length equal to or less than this one. On the other hand, the porosimetric curve is defined as the graph correlating the length of the void and the relative pore volume of the voids with a length equal to or less than this one.

**Figure 13:**
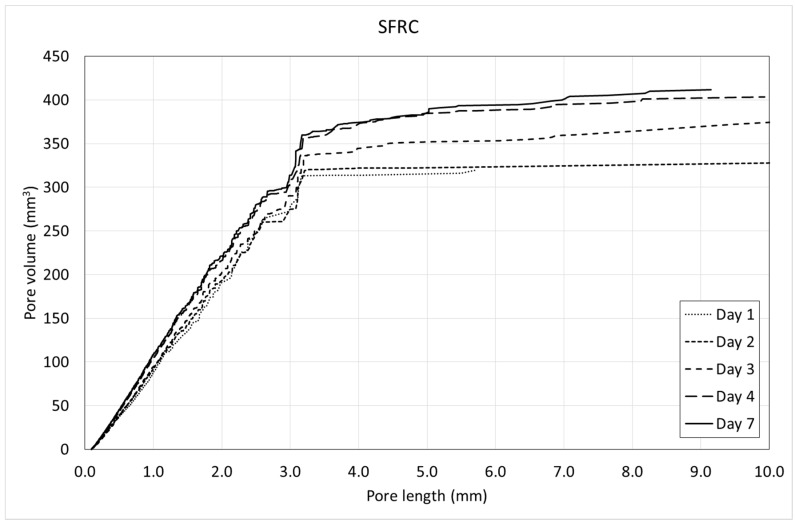
Pore volume curves of SFRC.

**Figure 14:**
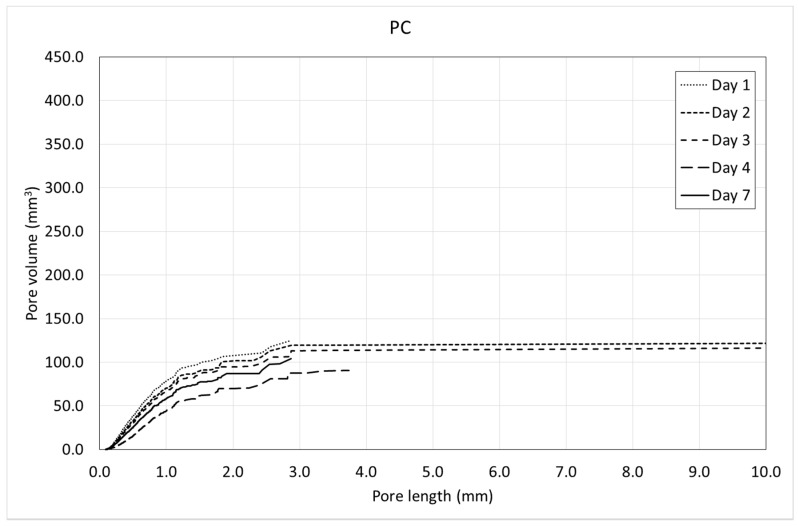
Pore volume curves of PC.

**Figure 15:**
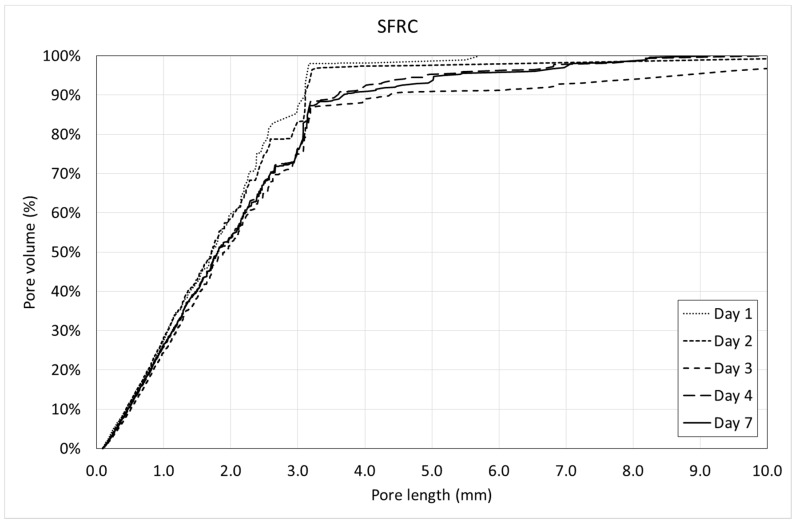
Porosimetric curves of SFRC.

**Figure 16:**
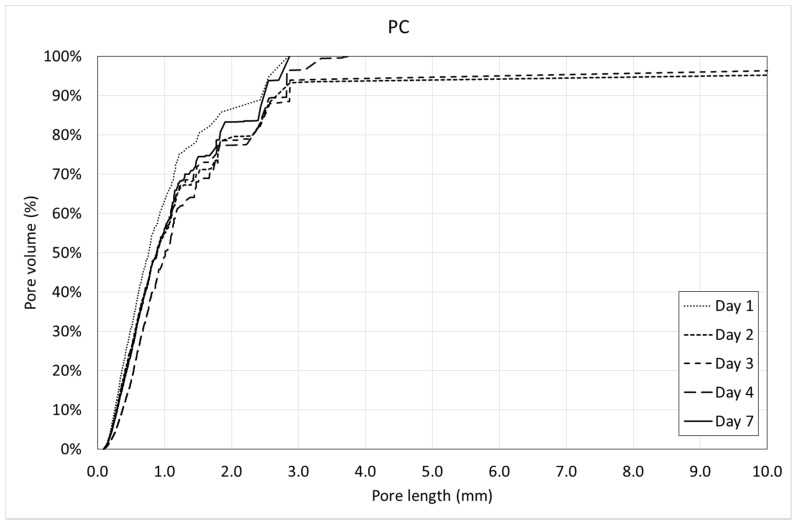
Porosimetric curves of PC.

Figure 13 and Figure 14 show the pore volume curves of the mixtures at the different ages. On the other side, Figure 15 and Figure 16 show the porosimetric curves of the mixtures at the different ages. In all cases, the maximum length is limited to 10 mm, since larger voids are residual.

Figure 13 and Figure 14 reveal that, in general, the pore volume curves show small variations with the age of concrete. In the case of SFRC, the curves belonging to the first days are placed below the ones belonging to the last days, being, in general, homothetic. This means that a progressive increase of the pore volume occurs with time, and it happens for all of the pore sizes.

In the case of PC, the curves belonging to the first days are placed over the ones belonging to the last days, being, in general, homothetic again. This means that a progressive decrease of the pore volume occurs with time, and it also happens for all of the pore sizes.

Figure 15 shows that, in general, the porosimetric curves belonging to the first days are placed over the ones belonging to the last days. This means that SFRC tends to increase the pore size with time.

In the case of PC (Figure 16), this tendency is not so clear. This means that PC tends to keep the pore size constant with time.

**Figure 17:**
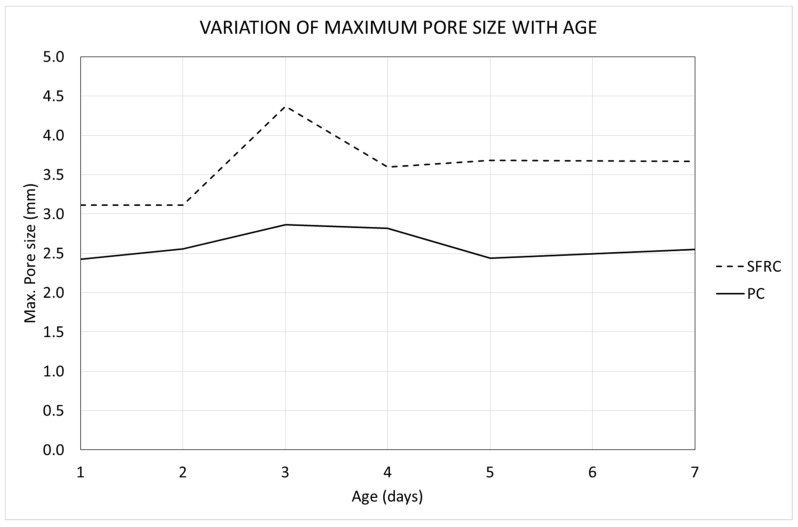
Variation of the nominal maximum pore size (NMPS) with the age.

An interesting parameter that can be obtained through the porosimetric curves is the nominal maximum pore size (NMPS), which can be defined, similarly to the well-known nominal maximum aggregate size (NMAS), as the pore length corresponding to a cumulative pore volume of 90%. This is a representative value of the pore size distribution, since the remaining 10% of the pore volume belongs to an extremely low number of individual pores (less than 0.05% of the pores, on average). Figure 17 show the variation of the NMPS in both mixtures with time.

Figure 17 reveals that the NMPS is greater in SFRC than in PC at all ages. This means that SFRC not only shows a greater pore volume (as can be observed in Figure 9), but also that the pores are bigger. In the case of SFRC, there is a progressive increase of the NMPS, which agrees with there being a progressive increase of the pore size with the age of concrete (Figure 15). On the contrary, in the case of PC, the curve shows a horizontal tendency, which means that there is not a variation (neither increase nor decrease) of the NMPS.

Both the pore volume curves (Figure 13 and Figure 14) and porosimetric curves (Figure 15 and Figure 16) reveal that, in the case of SFRC, the curves shows an initial part substantially straight up to a pore length of approximately 3 mm. This means that below a 3-mm pore length, there is a substantially uniform volume distribution. Beyond this critical pore length, the slope of the curves decreases drastically, becoming horizontal very quickly. The PC curves show a similar behavior, although the critical pore length can be established at 1 mm.

SFRC shows porosimetric curves flatter than the ones belonging to PC. This can be explained in terms of the stiffness of the cement matrix. In the case of SFRC, fibers provide additional stiffness to the cement paste, and they prevent the voids from collapsing. The greater the void, the greater the instability of the concrete. As a consequence, SFRC is able to withstand a greater percentage of larger voids than PC.

### 3.4. Variation of the Porosity and Porosimetric Curves along the Depth

**Figure 18:**
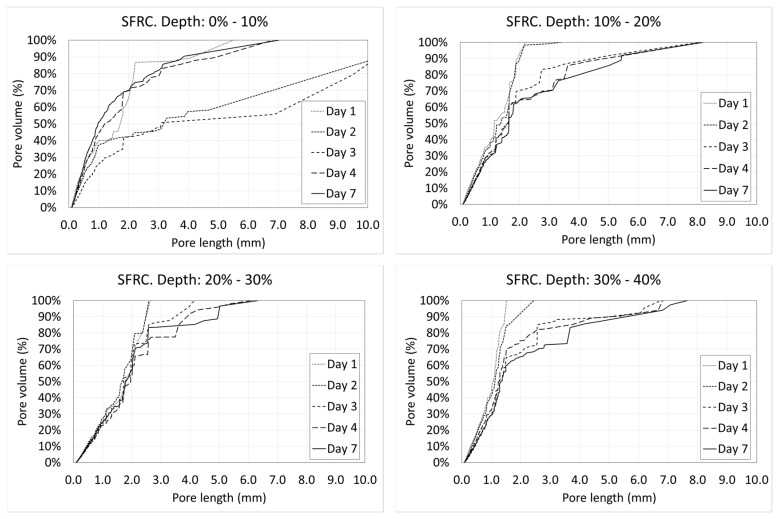

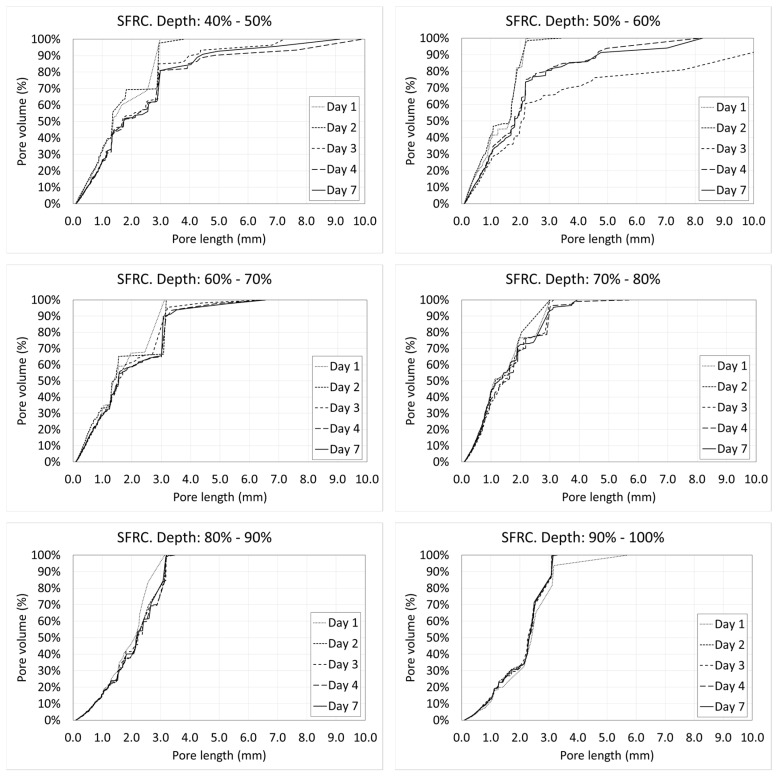
Porosimetric curves of SFRC at different ages and depths.

**Figure 19:**
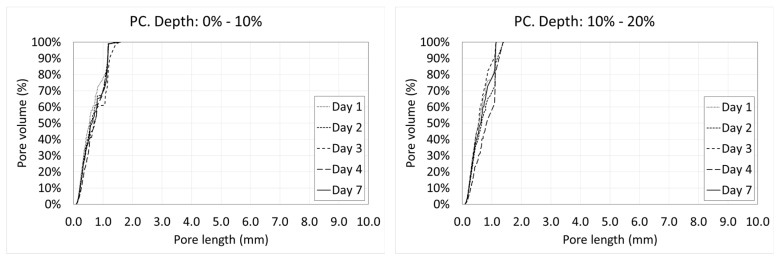

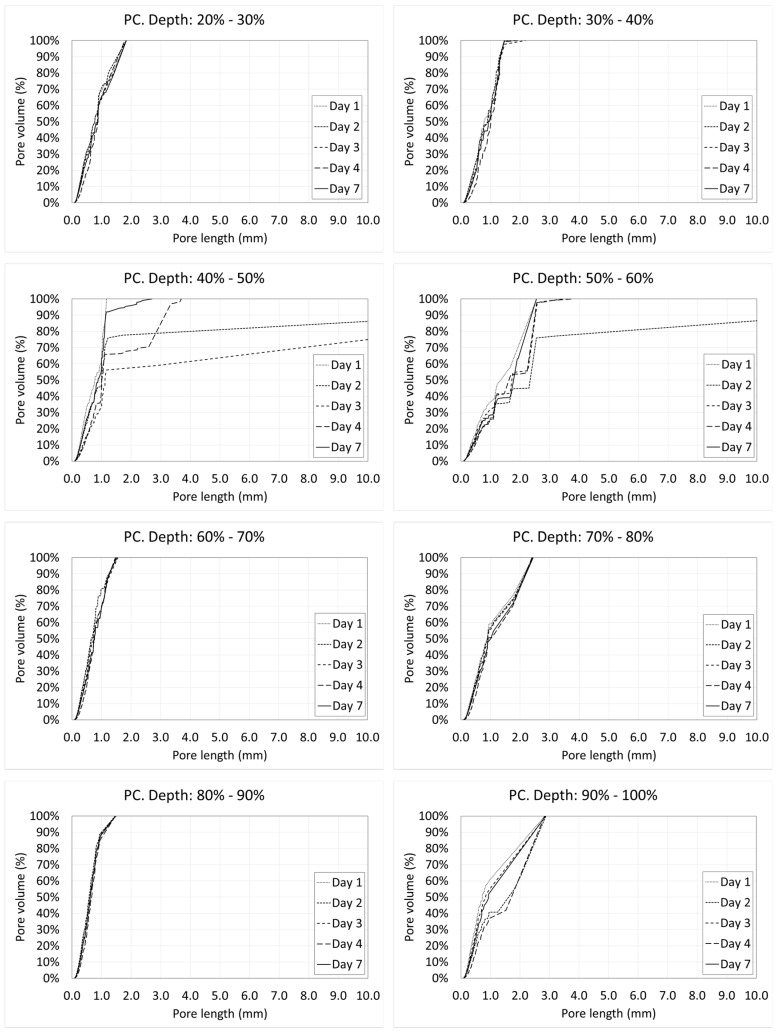
Porosimetric curves of PC at different ages and depths.

Voids are not uniformly distributed along the depth. The distance to the exposed surface, where the loss of water occurs, has a strong influence on the pore size distribution. Next, the porosimetric curves of all the days at the different depths are shown (Figure 18 and Figure 19).

In the case of SFRC (Figure 18), it can be observed that in the first two tenths of the depth, there is an intense variation of the porosity with the age of concrete. This is because of the water interchange with the environment (mainly evaporation). This activity starts to decrease beyond day four, showing almost identical graphs beyond this day.

Something similar can be observed at the middle height of the specimens. This is because the molds were filled in two parts, creating a small horizontal joint in this area.

Similarly to what happens in Figure 15, in the case of SFRC, all the curves are substantially bilinear. The first part is approximately a straight line with a big slope up to a critical pore length. Beyond this value, the slope of the curve decreases drastically. In all cases, lines belonging to the first part of the curve show a similar slope. However, the critical pore length varies with the depth, from 2 to 3 mm. This means that the percentage of greater pores decreases with the depth. It can be explained in terms of the hydrostatic pressure of fresh concrete, which increases with the depth. There is an inverse relation between the hydrostatic pressure of fresh concrete and the maximum stable void volume. Voids smaller than this critical volume are stable, but voids greater than that tend to collapse, becoming several smaller voids.

In the case of PC, a relevant temporary variation around the middle height of the specimens is observed. However, this phenomenon is not observed in the upper tenths. Except for the first day (where voids are full of free water and they are not detected by a CT scan), the tendency is toward a progressive reduction of the greater pores.

In this case, almost all of the curves are substantially straight. The slope of the curve is significantly greater than that in the case of SFRC, which is directly related to the viscosity and stiffness of the cement paste [46,47]. In this case, the lower stiffness of the cement paste of the PC causes the large pores to be much more unstable than in the case of SFRC, and they tend to collapse, becoming several smaller voids.

### 3.5. Shape Factor of the Voids

As explained before, the data provided by the CT scan and the DIP software lead us to know the volume and the length of each void. Using these two data, it is possible to obtain the shape factor of each pore, which is defined as the quotient between the volume of the void and the volume of the sphere circumscribed to the void, as shown in Equation (1) [49]:

(1) $$SF=\frac{V_{p}}{\frac{1}{6}\cdot \pi \cdot L_{p}{}^{3}}$$

where $V_{p}$ is the pore volume and $L_{p}$ is the pore length.

**Figure 20:**
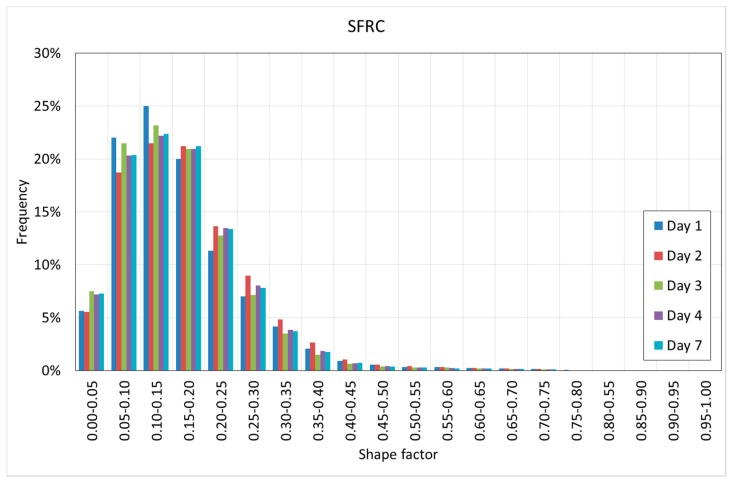
Histogram of the shape factor of SFRC.

**Figure 21:**
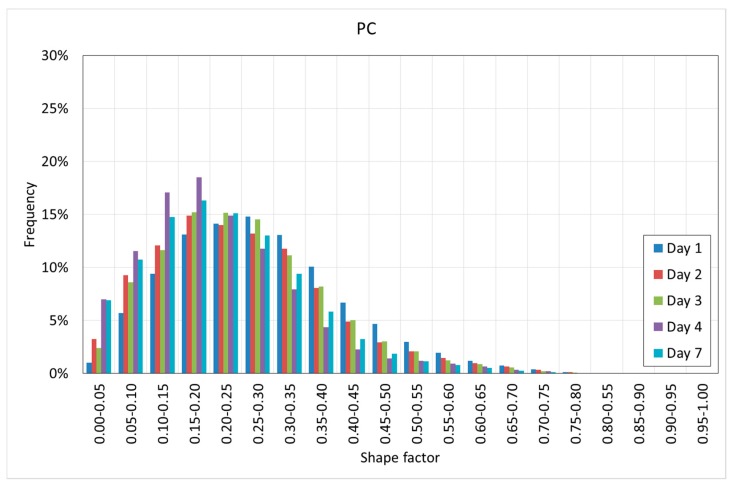
Histogram of the shape factor of PC.

Figure 20 and Figure 21 show the histograms of the shape factor of the different mixtures.

Figure 20 and Figure 21 reveal interesting results. First, it can be observed that in both cases, the voids are far from the spheres since in all of the cases, the shape factor is far from one.

SFRC shows smaller shape factors than PC, showing a mode value between 0.10–0.15, as well as more than 90% of the voids showing a shape factor below 0.30.

PC shows slightly greater shape factors, with a mode value between 0.15–0.25, as well as more than 90% of the voids showing a shape factor below 0.40.

Pores are the less stiff components of the cement paste, and they tend to occupy the space that is not occupied by the rest of the components of the cement paste. These ‘free’ spaces do not show spherical shapes; instead, they are flaky or elongated. In the case of SFRC, these spaces become even more elongated because of the fibers. Again, fibers modify the pore morphology.

**Figure 22:**
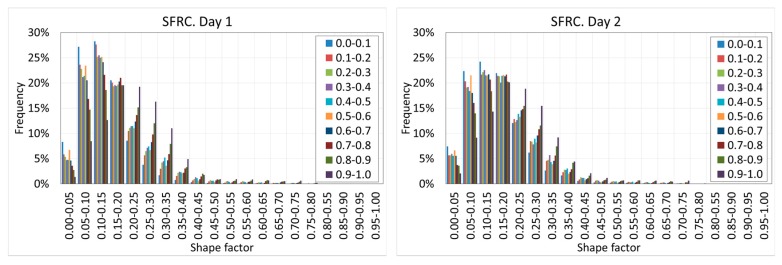

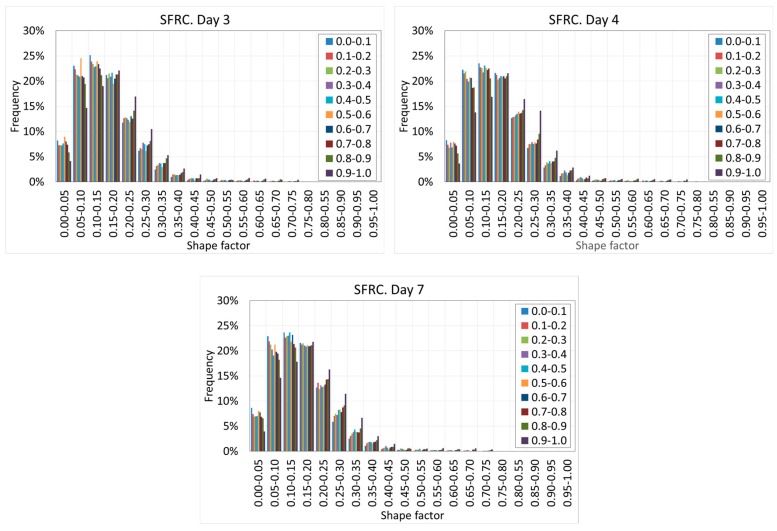
Histograms of the shape factors of SFRC at different depths and ages.

**Figure 23:**
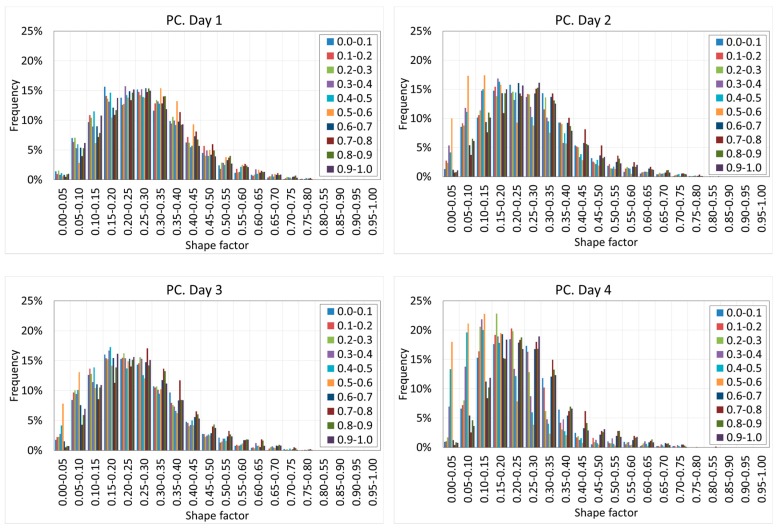

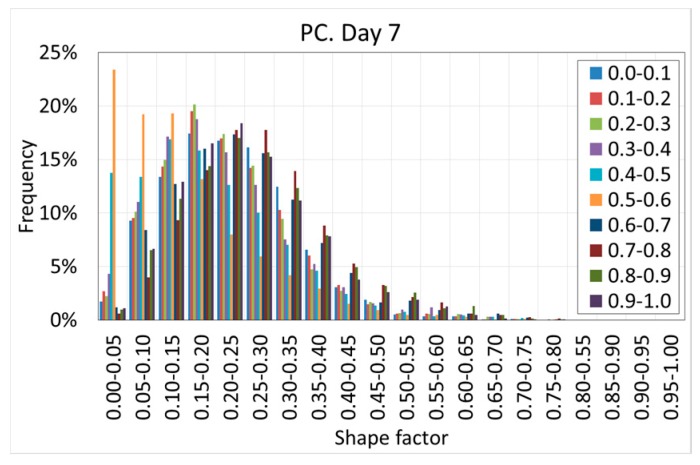
Histograms of the shape factors of PC at different depths and ages.

The shape factor shows relevant variations along the depth. Figure 22 and Figure 23 show the histograms of the shape factor at different ages of concrete and at different depths for both SFRC and PC.

Figure 22 and Figure 23 reveal that in general, the shape of the histograms remains constant with the age of the concrete, which demonstrates that the shape of the voids does not vary with time.

However, a substantial variation of the shape of the histograms at different depths is observed. When the depth increases, the histograms moves toward the right. This phenomenon can be observed on all the days, although it is more intense at the earliest ages. This is because the increase of the hydrostatic pressure of the fresh concrete promotes more spherical voids, which are more stable under higher hydrostatic pressure values.

This variation cannot be clearly shown in the case of PC because of the collapse of the voids due to the lower stiffness of the cement paste, which redraws the shape of the voids. This can be clearly observed on day four, when a substantial increase of the elongated voids was observed.

## 4. Conclusions

This paper analyzed the internal pore morphology of two different mixtures through the use of CT scan technology. Both of them had the same matrix (same type and amount of cement, fine aggregate, nanosilica, water, and superplasticizer); the only difference was that one mixture, which was called SFRC, included steel fibers by 0.1% volume, while the other mixture, which was called PC, did not include fibers. The pore morphology was measured during the first week (when most of the curing process occurs) at five different ages, i.e., one day, two days, three days, four days, and seven days.

All of the specimens were kept in a curing room at 20 °C and 100% humidity. The information provided by the CT scan was post-processed using DIP software. Each individual void was identified and isolated, and its main parameters were extracted, which were the X, Y, and Z coordinates of the center of gravity, the volume, and the length, respectively.

Some worthy and interesting results are summarized below:

- The SFRC specimens showed greater porosity than the PC ones. This difference can be observed on all the studied days. Moreover, the porosity increased with the age of the concrete in the case of SFRC, while the porosity remained almost constant in the case of PC.
- Regarding the spatial distribution of the porosity, a progressive increase of the porosity with the depth was observed in all of the cases. This was especially clear in the case of SFRC, but this tendency was not so clear in the case of PC. However, in both cases, the lowest porosity was observed in the first two tenths of the specimens.
- The stiffness of the cement paste was behind most of the observed behaviors. Fibers provided additional stiffness to the cement paste, which allowed it to retain porosity. In the case of PC, where there are no fibers, the stiffness of the fresh concrete paste was small and the voids, especially the big ones, tended to collapse and the air tended to leave the concrete.
- Water loss happens more quickly when it is closer to the exposed surface. Not all of it is used to create cement paste, because some of it evaporates. The consequence is that when concrete is closer to the exposed surface, less stiffness and a smaller amount of voids are retained. Even in the case of SFRC, fibers do not provide the extra stiffness in an efficient way.
- The porosimetric curves showed two different stages. The first one belonged to the smaller sizes, from zero to a critical length, where the curves showed a straight line. Beyond this critical length, the slope of the curves decreased drastically. In the case of SFRC, the critical length could be defined as 3 mm, while in the case of PC, the critical length was 1 mm. This behavior could be observed along the depth, although the value of the critical length varied slightly with the depth, especially in the case of SFRC.
- The critical length is, again, strongly related to the stiffness of the fresh cement paste and its capacity to create voids. In the case of SFRC, the extra stiffness provided by the fibers allowed the concrete to retain larger pores up to 3 mm in length. Beyond this value, the pores became more unstable and tended to collapse. In the case of PC, cement paste is less stiff, and the capacity to retain pores is reduced; voids beyond 1 mm in length seem to be difficult to retain, and tend to collapse.
- The fibers changed the concrete porosity through two mechanisms. The first one was to trap air during the concreting process and also increase the viscosity of the mixture, which complicated the expelling of the trapped air during the concreting. The second was to provide an extra stiffness to avoid the collapse of the voids due to water loss.
- The histograms of shape factors showed that the voids were far from spheres, i.e., they were more flaky or elongated. SFRC showed a shape factor smaller than the one shown by PC, which was due to the presence of fibers. In this case, the variation of the shape factor with the age of concrete was insignificant. On the contrary, the shape factor increased with the depth.

The modification of the pore morphology caused by the fiber can potentially affect the macroscopic response of hardened concrete, especially in those properties more directly related to porosity, such as frost resistance, fire resistance, creep, shrinkage, and fatigue, among others.
